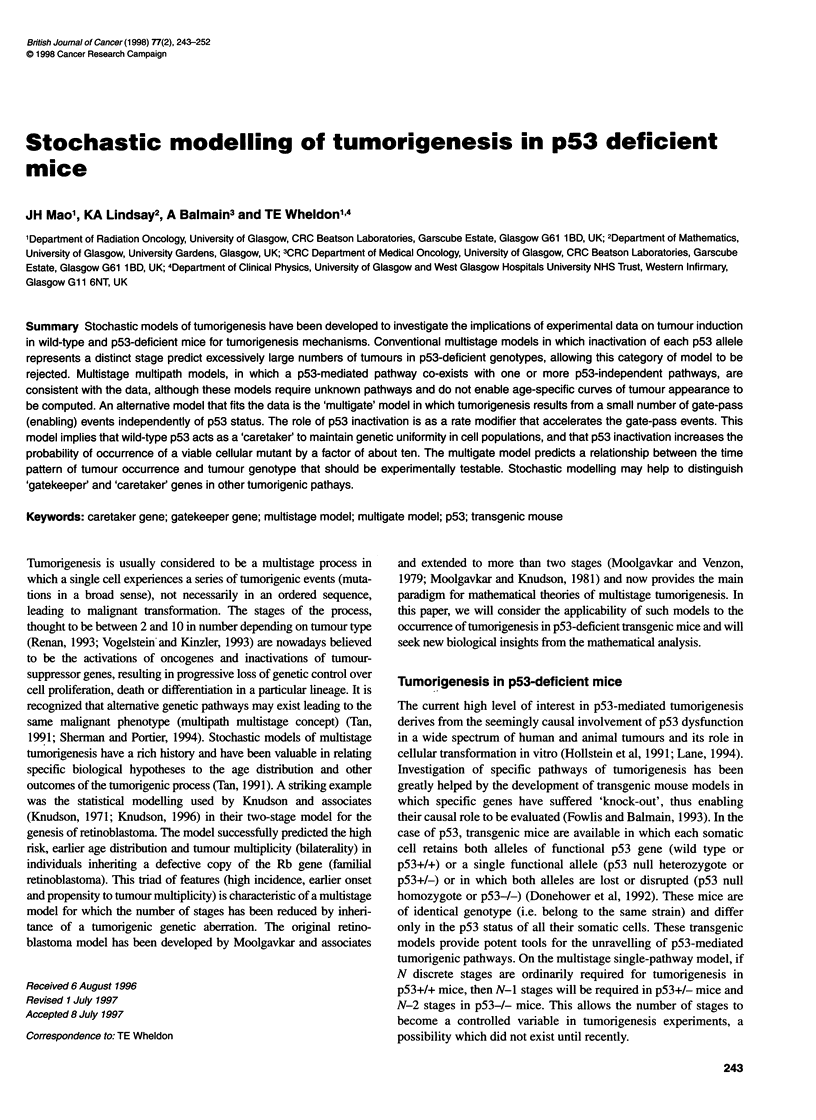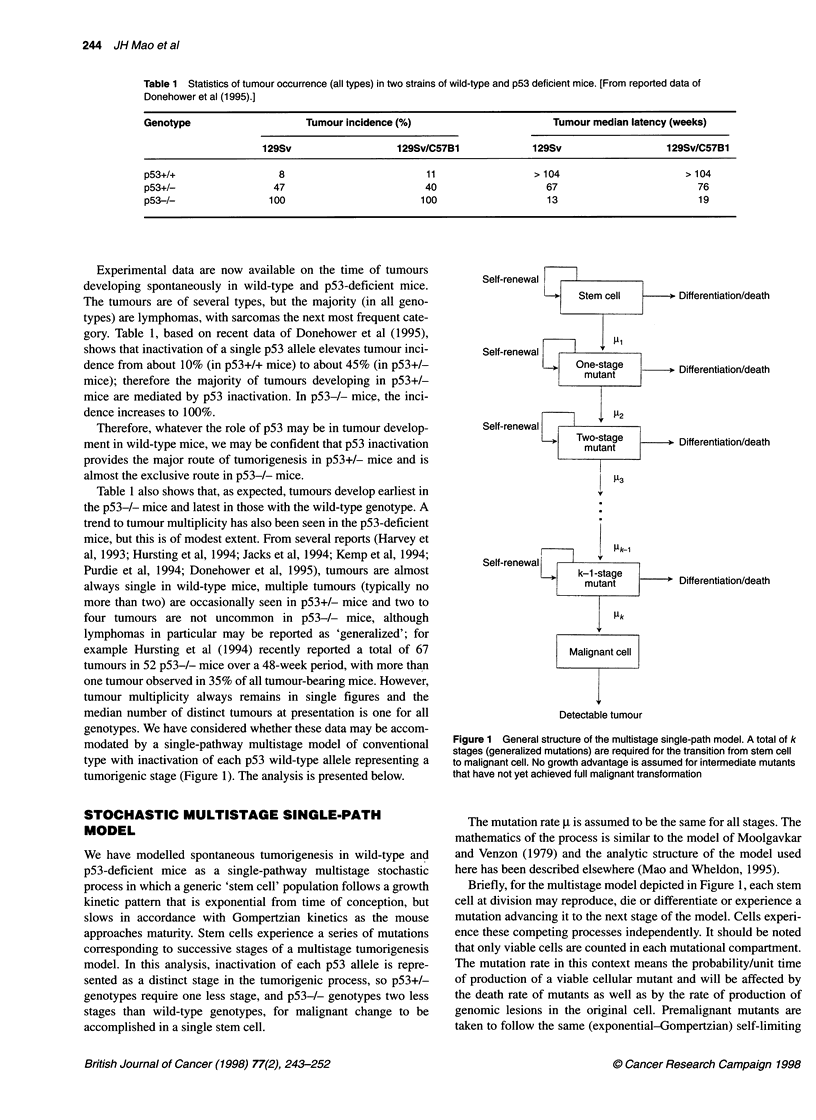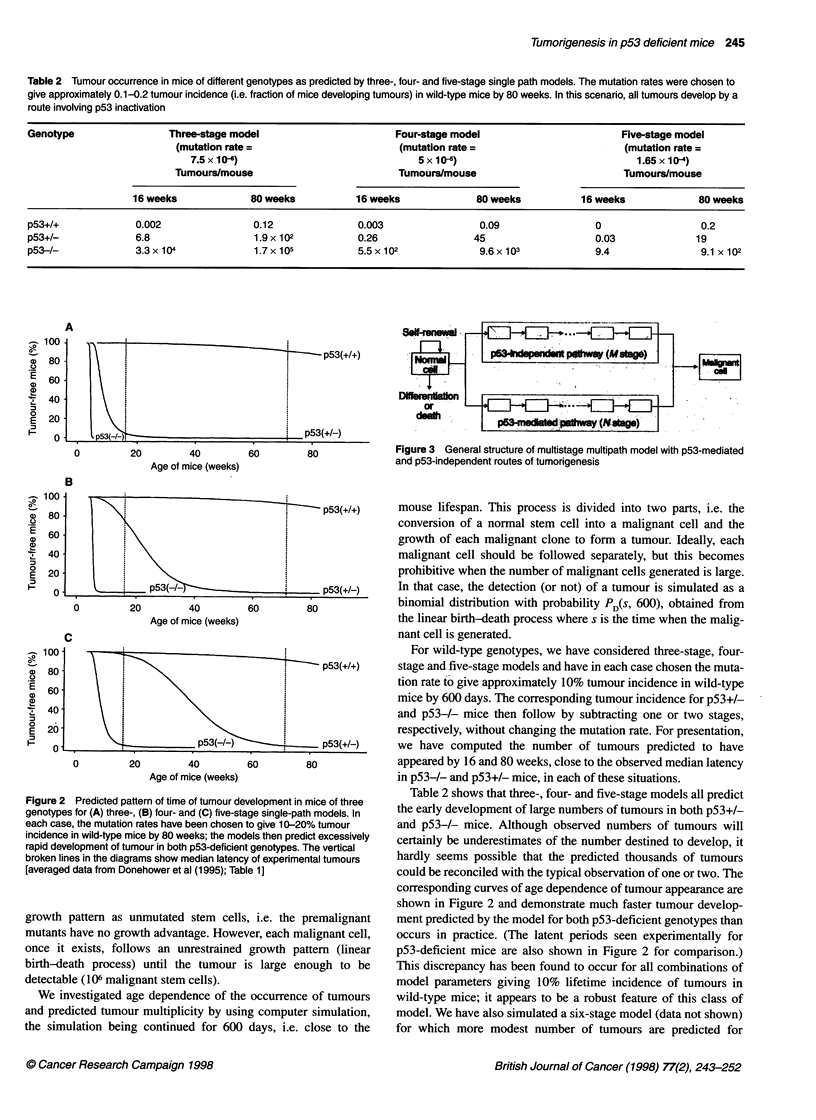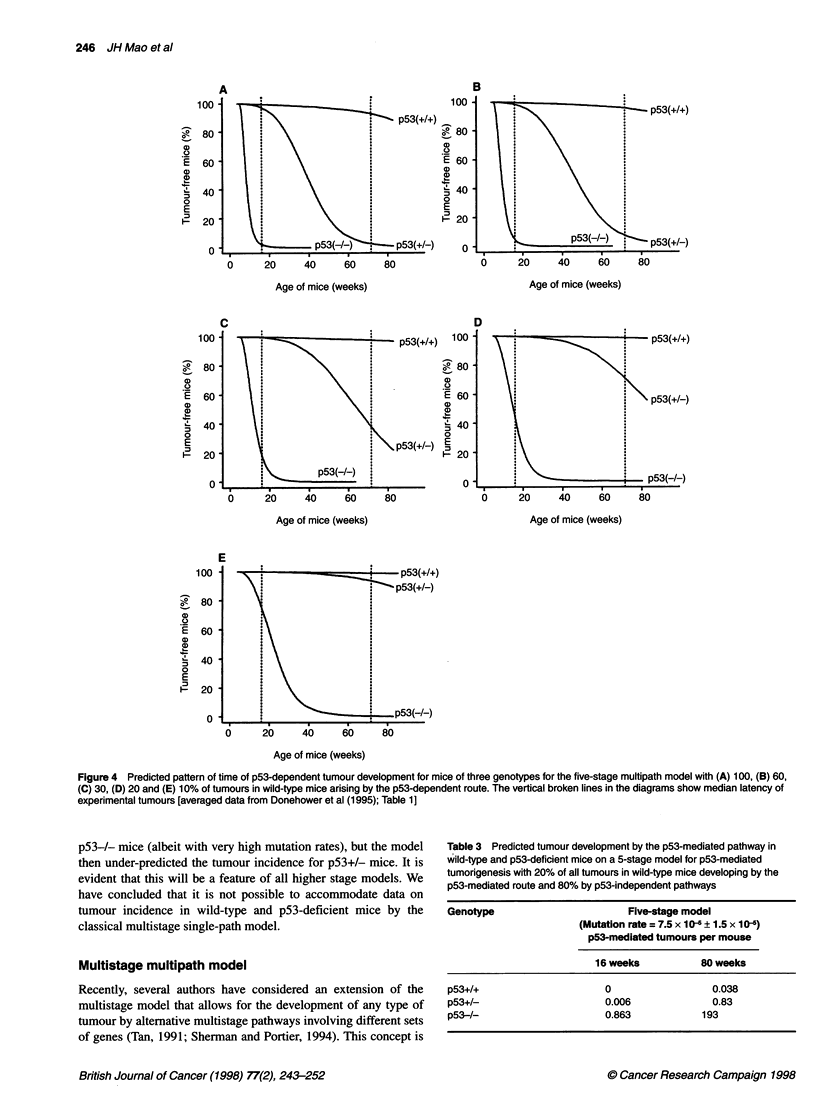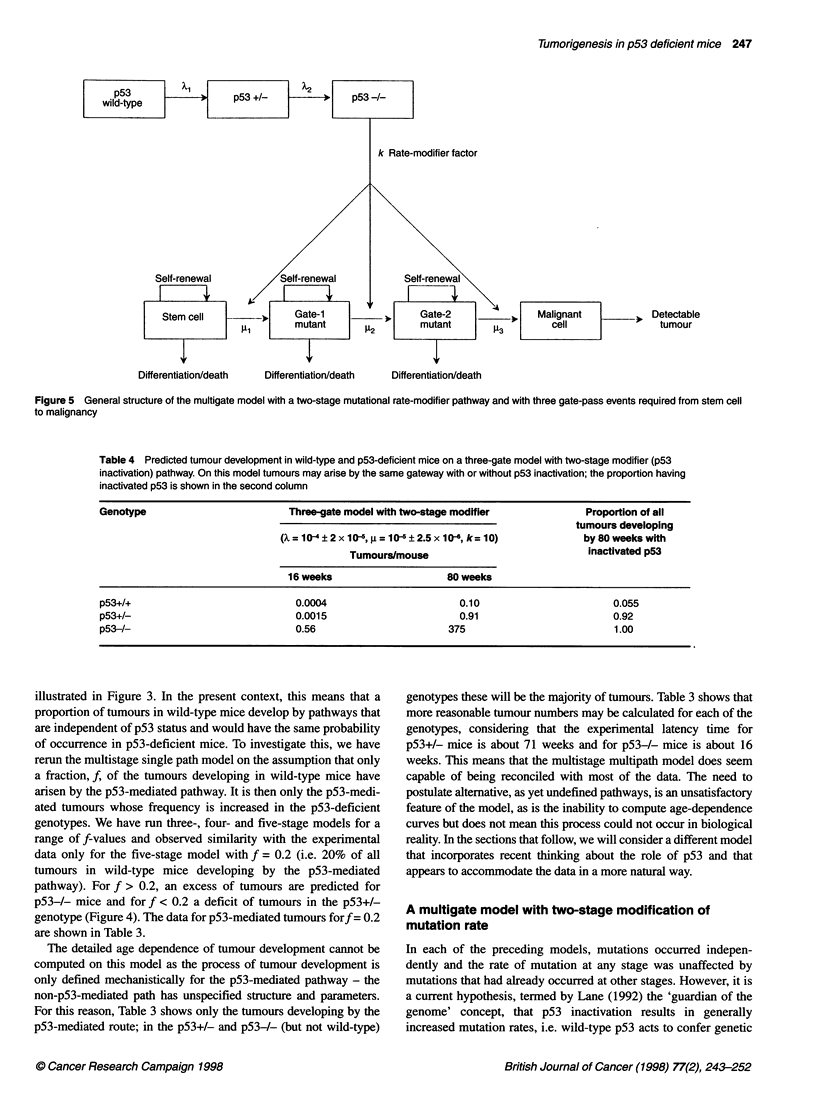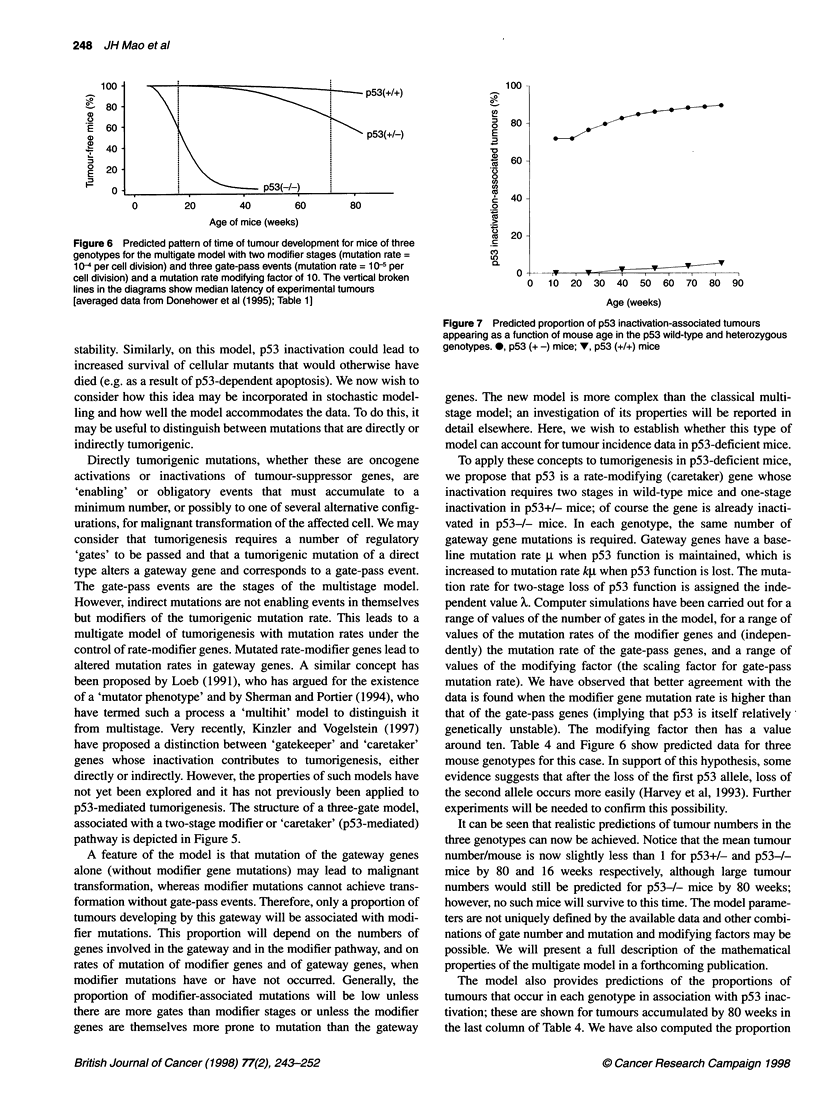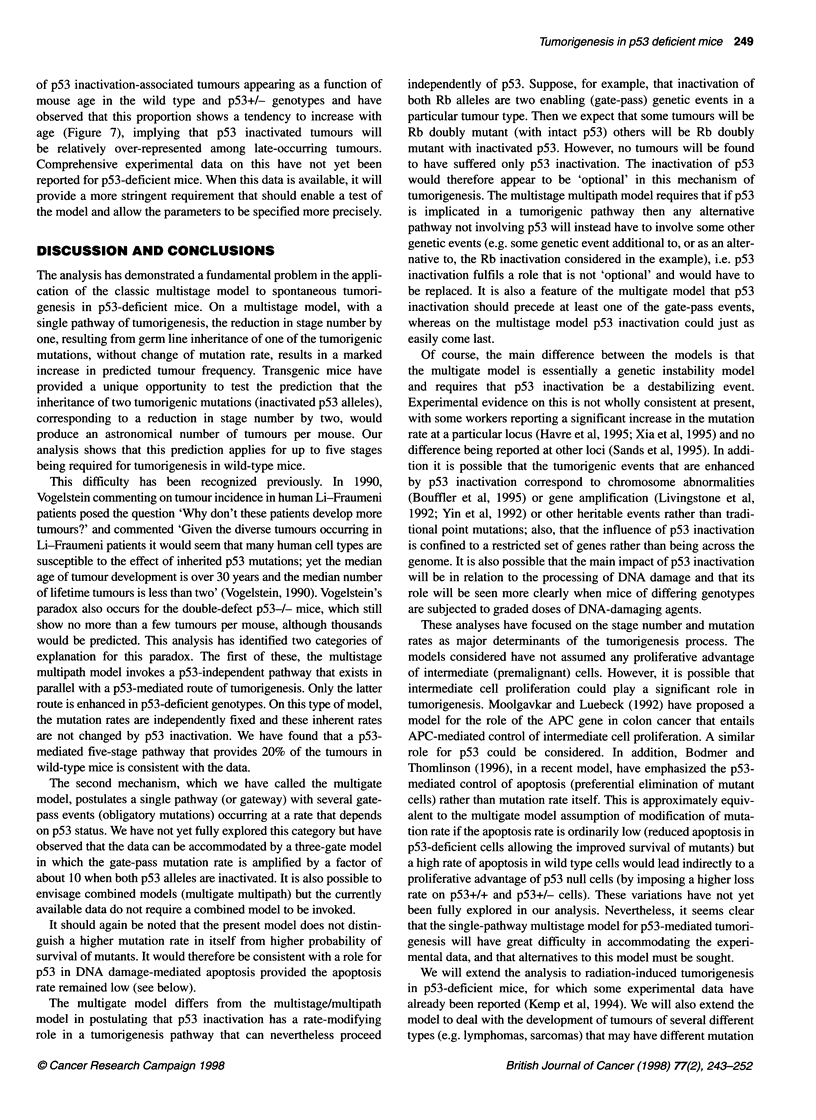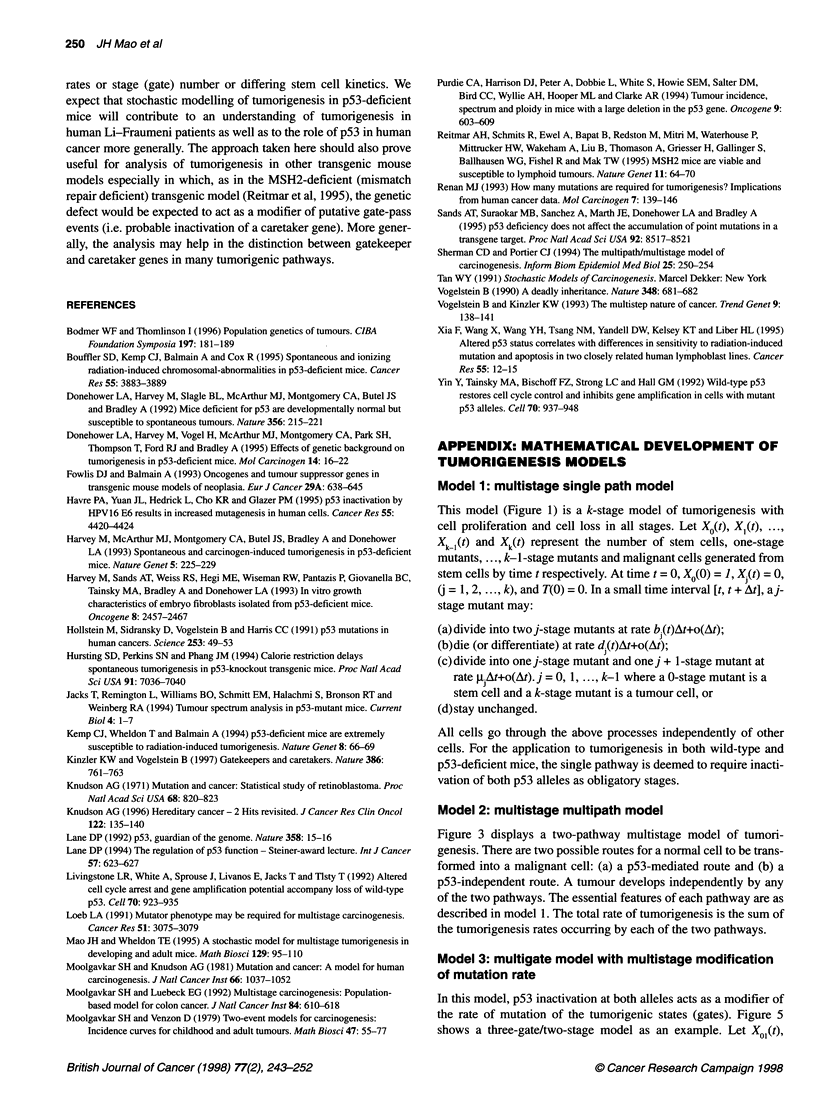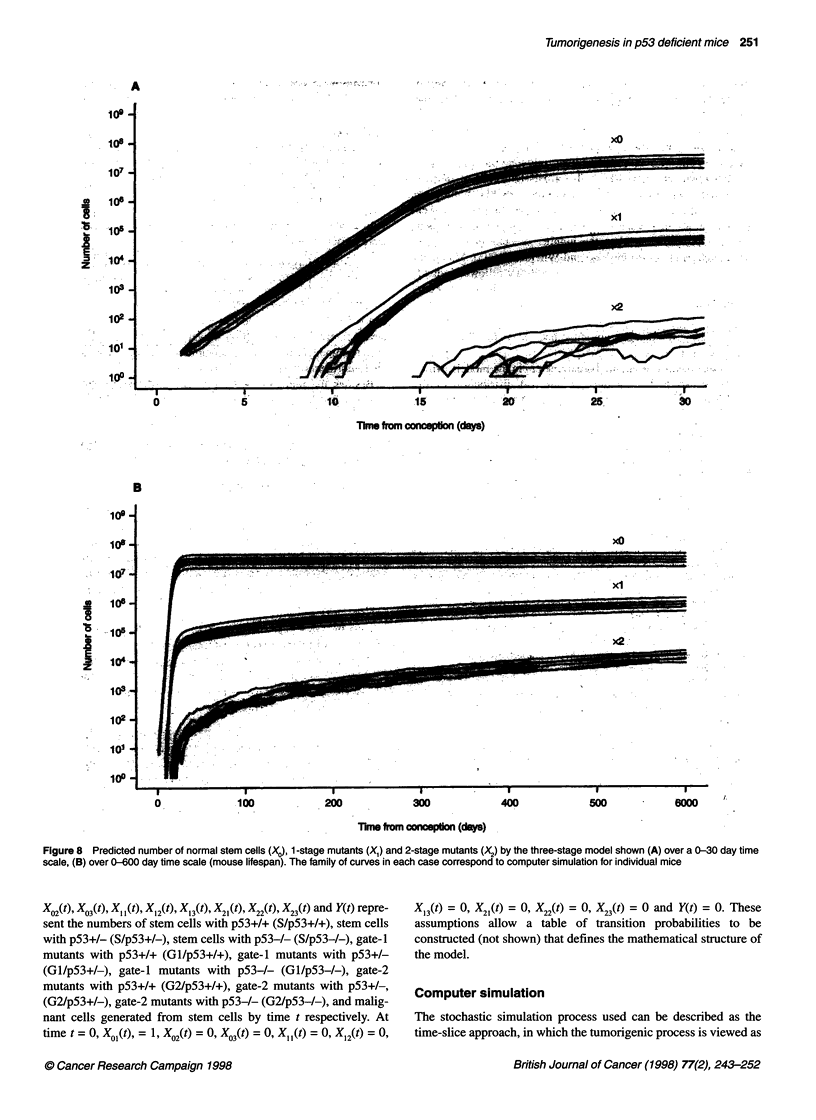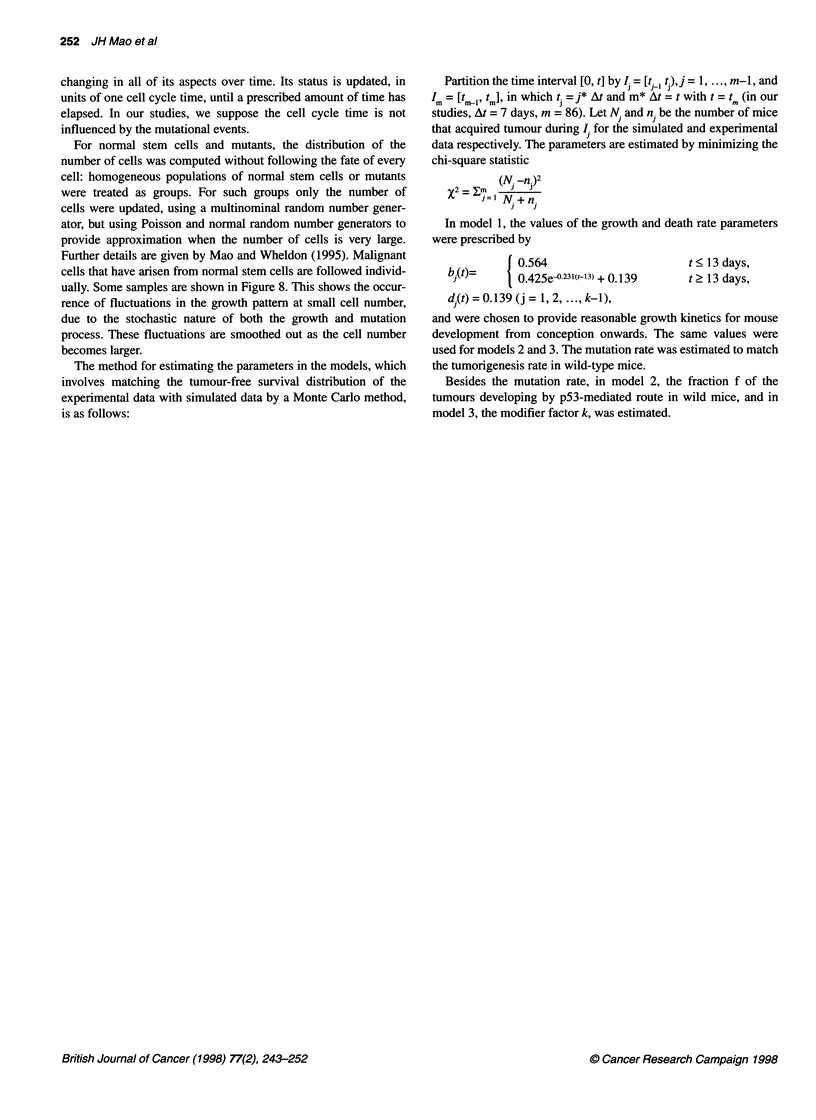# Stochastic modelling of tumorigenesis in p53 deficient mice.

**DOI:** 10.1038/bjc.1998.40

**Published:** 1998

**Authors:** J. H. Mao, K. A. Lindsay, A. Balmain, T. E. Wheldon

**Affiliations:** Department of Radiation Oncology, University of Glasgow, CRC Beatson Laboratories, UK.

## Abstract

Stochastic models of tumorigenesis have been developed to investigate the implications of experimental data on tumour induction in wild-type and p53-deficient mice for tumorigenesis mechanisms. Conventional multistage models in which inactivation of each p53 allele represents a distinct stage predict excessively large numbers of tumours in p53-deficient genotypes, allowing this category of model to be rejected. Multistage multipath models, in which a p53-mediated pathway co-exists with one or more p53-independent pathways, are consistent with the data, although these models require unknown pathways and do not enable age-specific curves of tumour appearance to be computed. An alternative model that fits the data is the 'multigate' model in which tumorigenesis results from a small number of gate-pass (enabling) events independently of p53 status. The role of p53 inactivation is as a rate modifier that accelerates the gate-pass events. This model implies that wild-type p53 acts as a 'caretaker' to maintain genetic uniformity in cell populations, and that p53 inactivation increases the probability of occurrence of a viable cellular mutant by a factor of about ten. The multigate model predicts a relationship between the time pattern of tumour occurrence and tumour genotype that should be experimentally testable. Stochastic modelling may help to distinguish 'gatekeeper' and 'caretaker' genes in other tumorigenic pathays.


British Journal of Cancer (1998) 77(2), 243–252
© 1998 Cancer Research Campaign

### Stochastic modelling of tumorigenesis in p53 deficient mice

**JH Mao[1], KA Lindsay[2], A Balmain[3] and TE Wheldon[1,4]**

[1]Department of Radiation Oncology, University of Glasgow, CRC Beatson Laboratories, Garscube Estate, Glasgow G61 1BD, UK; [2]Department of Mathematics, University of Glasgow, University Gardens, Glasgow, UK; [3]CRC Department of Medical Oncology, University of Glasgow, CRC Beatson Laboratories, Garscube Estate, Glasgow G61 1BD, UK; [4]Department of Clinical Physics, University of Glasgow and West Glasgow Hospitals University NHS Trust, Western Infirmary, Glasgow G11 6NT, UK

**Summary** Stochastic models of tumorigenesis have been developed to investigate the implications of experimental data on tumour induction in wild-type and p53-deficient mice for tumorigenesis mechanisms. Conventional multistage models in which inactivation of each p53 allele represents a distinct stage predict excessively large numbers of tumours in p53-deficient genotypes, allowing this category of model to be rejected. Multistage multipath models, in which a p53-mediated pathway co-exists with one or more p53-independent pathways, are consistent with the data, although these models require unknown pathways and do not enable age-specific curves of tumour appearance to be computed. An alternative model that fits the data is the 'multigate' model in which tumorigenesis results from a small number of gate-pass (enabling) events independently of p53 status. The role of p53 inactivation is as a rate modifier that accelerates the gate-pass events. This model implies that wild-type p53 acts as a 'caretaker' to maintain genetic uniformity in cell populations, and that p53 inactivation increases the probability of occurrence of a viable cellular mutant by a factor of about ten. The multigate model predicts a relationship between the time pattern of tumour occurrence and tumour genotype that should be experimentally testable. Stochastic modelling may help to distinguish 'gatekeeper' and 'caretaker' genes in other tumorigenic pathays.

**Keywords:** caretaker gene; gatekeeper gene; multistage model; multigate model; p53; transgenic mouse

Tumorigenesis is usually considered to be a multistage process in which a single cell experiences a series of tumorigenic events (mutations in a broad sense), not necessarily in an ordered sequence, leading to malignant transformation. The stages of the process, thought to be between 2 and 10 in number depending on tumour type (Renan, 1993; Vogelstein and Kinzler, 1993) are nowadays believed to be the activations of oncogenes and inactivations of tumour-suppressor genes, resulting in progressive loss of genetic control over cell proliferation, death or differentiation in a particular lineage. It is recognized that alternative genetic pathways may exist leading to the same malignant phenotype (multipath multistage concept) (Tan, 1991; Sherman and Portier, 1994). Stochastic models of multistage tumorigenesis have a rich history and have been valuable in relating specific biological hypotheses to the age distribution and other outcomes of the tumorigenic process (Tan, 1991). A striking example was the statistical modelling used by Knudson and associates (Knudson, 1971; Knudson, 1996) in their two-stage model for the genesis of retinoblastoma. The model successfully predicted the high risk, earlier age distribution and tumour multiplicity (bilaterality) in individuals inheriting a defective copy of the Rb gene (familial retinoblastoma). This triad of features (high incidence, earlier onset and propensity to tumour multiplicity) is characteristic of a multistage model for which the number of stages has been reduced by inheritance of a tumorigenic genetic aberration. The original retinoblastoma model has been developed by Moolgavkar and associates and extended to more than two stages (Moolgavkar and Venzon, 1979; Moolgavkar and Knudson, 1981) and now provides the main paradigm for mathematical theories of multistage tumorigenesis. In this paper, we will consider the applicability of such models to the occurrence of tumorigenesis in p53-deficient transgenic mice and will seek new biological insights from the mathematical analysis.

*Received 6 August 1996*
*Revised 1 July 1997*
*Accepted 8 July 1997*

*Correspondence to:* TE Wheldon

### Tumorigenesis in p53-deficient mice

The current high level of interest in p53-mediated tumorigenesis derives from the seemingly causal involvement of p53 dysfunction in a wide spectrum of human and animal tumours and its role in cellular transformation in vitro (Hollstein et al, 1991; Lane, 1994). Investigation of specific pathways of tumorigenesis has been greatly helped by the development of transgenic mouse models in which specific genes have suffered 'knock-out', thus enabling their causal role to be evaluated (Fowlis and Balmain, 1993). In the case of p53, transgenic mice are available in which each somatic cell retains both alleles of functional p53 gene (wild type or p53+/+) or a single functional allele (p53 null heterozygote or p53+/–) or in which both alleles are lost or disrupted (p53 null homozygote or p53–/–) (Donehower et al, 1992). These mice are of identical genotype (i.e. belong to the same strain) and differ only in the p53 status of all their somatic cells. These transgenic models provide potent tools for the unravelling of p53-mediated tumorigenic pathways. On the multistage single-pathway model, if $N$ discrete stages are ordinarily required for tumorigenesis in p53+/+ mice, then $N$–1 stages will be required in p53+/– mice and $N$–2 stages in p53–/– mice. This allows the number of stages to become a controlled variable in tumorigenesis experiments, a possibility which did not exist until recently.

**Table 1** Statistics of tumour occurrence (all types) in two strains of wild-type and p53 deficient mice. [From reported data of Donehower et al (1995).]

| Genotype | Tumour incidence (%) | | Tumour median latency (weeks) | |
|---|---|---|---|---|
| | 129Sv | 129Sv/C57B1 | 129Sv | 129Sv/C57B1 |
| p53+/+ | 8 | 11 | > 104 | > 104 |
| p53+/– | 47 | 40 | 67 | 76 |
| p53–/– | 100 | 100 | 13 | 19 |

Experimental data are now available on the time of tumours developing spontaneously in wild-type and p53-deficient mice. The tumours are of several types, but the majority (in all genotypes) are lymphomas, with sarcomas the next most frequent category. Table 1, based on recent data of Donehower et al (1995), shows that inactivation of a single p53 allele elevates tumour incidence from about 10% (in p53+/+ mice) to about 45% (in p53+/– mice); therefore the majority of tumours developing in p53+/– mice are mediated by p53 inactivation. In p53–/– mice, the incidence increases to 100%.

Therefore, whatever the role of p53 may be in tumour development in wild-type mice, we may be confident that p53 inactivation provides the major route of tumorigenesis in p53+/– mice and is almost the exclusive route in p53–/– mice.

Table 1 also shows that, as expected, tumours develop earliest in the p53–/– mice and latest in those with the wild-type genotype. A trend to tumour multiplicity has also been seen in the p53-deficient mice, but this is of modest extent. From several reports (Harvey et al, 1993; Hursting et al, 1994; Jacks et al, 1994; Kemp et al, 1994; Purdie et al, 1994; Donehower et al, 1995), tumours are almost always single in wild-type mice, multiple tumours (typically no more than two) are occasionally seen in p53+/– mice and two to four tumours are not uncommon in p53–/– mice, although lymphomas in particular may be reported as 'generalized'; for example Hursting et al (1994) recently reported a total of 67 tumours in 52 p53–/– mice over a 48-week period, with more than one tumour observed in 35% of all tumour-bearing mice. However, tumour multiplicity always remains in single figures and the median number of distinct tumours at presentation is one for all genotypes. We have considered whether these data may be accommodated by a single-pathway multistage model of conventional type with inactivation of each p53 wild-type allele representing a tumorigenic stage (Figure 1). The analysis is presented below.

**Figure 1** General structure of the multistage single-path model. A total of *k* stages (generalized mutations) are required for the transition from stem cell to malignant cell. No growth advantage is assumed for intermediate mutants that have not yet achieved full malignant transformation

### STOCHASTIC MULTISTAGE SINGLE-PATH MODEL

We have modelled spontaneous tumorigenesis in wild-type and p53-deficient mice as a single-pathway multistage stochastic process in which a generic 'stem cell' population follows a growth kinetic pattern that is exponential from time of conception, but slows in accordance with Gompertzian kinetics as the mouse approaches maturity. Stem cells experience a series of mutations corresponding to successive stages of a multistage tumorigenesis model. In this analysis, inactivation of each p53 allele is represented as a distinct stage in the tumorigenic process, so p53+/– genotypes require one less stage, and p53–/– genotypes two less stages than wild-type genotypes, for malignant change to be accomplished in a single stem cell.

The mutation rate $\mu$ is assumed to be the same for all stages. The mathematics of the process is similar to the model of Moolgavkar and Venzon (1979) and the analytic structure of the model used here has been described elsewhere (Mao and Wheldon, 1995).

Briefly, for the multistage model depicted in Figure 1, each stem cell at division may reproduce, die or differentiate or experience a mutation advancing it to the next stage of the model. Cells experience these competing processes independently. It should be noted that only viable cells are counted in each mutational compartment. The mutation rate in this context means the probability/unit time of production of a viable cellular mutant and will be affected by the death rate of mutants as well as by the rate of production of genomic lesions in the original cell. Premalignant mutants are taken to follow the same (exponential–Gompertzian) self-limiting

 © Cancer Research Campaign 1998

**Table 2** Tumour occurrence in mice of different genotypes as predicted by three-, four- and five-stage single path models. The mutation rates were chosen to give approximately 0.1–0.2 tumour incidence (i.e. fraction of mice developing tumours) in wild-type mice by 80 weeks. In this scenario, all tumours develop by a route involving p53 inactivation

| Genotype | Three-stage model (mutation rate = $7.5 \times 10^{-6}$) Tumours/mouse | | Four-stage model (mutation rate = $5 \times 10^{-5}$) Tumours/mouse | | Five-stage model (mutation rate = $1.65 \times 10^{-4}$) Tumours/mouse | |
|---|---|---|---|---|---|---|
| | 16 weeks | 80 weeks | 16 weeks | 80 weeks | 16 weeks | 80 weeks |
| p53+/+ | 0.002 | 0.12 | 0.003 | 0.09 | 0 | 0.2 |
| p53+/– | 6.8 | $1.9 \times 10^2$ | 0.26 | 45 | 0.03 | 19 |
| p53–/– | $3.3 \times 10^4$ | $1.7 \times 10^5$ | $5.5 \times 10^2$ | $9.6 \times 10^3$ | 9.4 | $9.1 \times 10^2$ |

**Figure 2** Predicted pattern of time of tumour development in mice of three genotypes for (**A**) three-, (**B**) four- and (**C**) five-stage single-path models. In each case, the mutation rates have been chosen to give 10–20% tumour incidence in wild-type mice by 80 weeks; the models then predict excessively rapid development of tumour in both p53-deficient genotypes. The vertical broken lines in the diagrams show median latency of experimental tumours [averaged data from Donehower et al (1995); Table 1]

growth pattern as unmutated stem cells, i.e. the premalignant mutants have no growth advantage. However, each malignant cell, once it exists, follows an unrestrained growth pattern (linear birth–death process) until the tumour is large enough to be detectable ($10^6$ malignant stem cells).

We investigated age dependence of the occurrence of tumours and predicted tumour multiplicity by using computer simulation, the simulation being continued for 600 days, i.e. close to the mouse lifespan. This process is divided into two parts, i.e. the conversion of a normal stem cell into a malignant cell and the growth of each malignant clone to form a tumour. Ideally, each malignant cell should be followed separately, but this becomes prohibitive when the number of malignant cells generated is large. In that case, the detection (or not) of a tumour is simulated as a binomial distribution with probability $P_D(s, 600)$, obtained from the linear birth–death process where $s$ is the time when the malignant cell is generated.

**Figure 3** General structure of multistage multipath model with p53-mediated and p53-independent routes of tumorigenesis

For wild-type genotypes, we have considered three-stage, four-stage and five-stage models and have in each case chosen the mutation rate to give approximately 10% tumour incidence in wild-type mice by 600 days. The corresponding tumour incidence for p53+/– and p53–/– mice then follow by subtracting one or two stages, respectively, without changing the mutation rate. For presentation, we have computed the number of tumours predicted to have appeared by 16 and 80 weeks, close to the observed median latency in p53–/– and p53+/– mice, in each of these situations.

Table 2 shows that three-, four- and five-stage models all predict the early development of large numbers of tumours in both p53+/– and p53–/– mice. Although observed numbers of tumours will certainly be underestimates of the number destined to develop, it hardly seems possible that the predicted thousands of tumours could be reconciled with the typical observation of one or two. The corresponding curves of age dependence of tumour appearance are shown in Figure 2 and demonstrate much faster tumour development predicted by the model for both p53-deficient genotypes than occurs in practice. (The latent periods seen experimentally for p53-deficient mice are also shown in Figure 2 for comparison.) This discrepancy has been found to occur for all combinations of model parameters giving 10% lifetime incidence of tumours in wild-type mice; it appears to be a robust feature of this class of model. We have also simulated a six-stage model (data not shown) for which more modest number of tumours are predicted for

© Cancer Research Campaign 1998

**Figure 4** Predicted pattern of time of p53-dependent tumour development for mice of three genotypes for the five-stage multipath model with (**A**) 100, (**B**) 60, (**C**) 30, (**D**) 20 and (**E**) 10% of tumours in wild-type mice arising by the p53-dependent route. The vertical broken lines in the diagrams show median latency of experimental tumours [averaged data from Donehower et al (1995); Table 1]

p53–/– mice (albeit with very high mutation rates), but the model then under-predicted the tumour incidence for p53+/– mice. It is evident that this will be a feature of all higher stage models. We have concluded that it is not possible to accommodate data on tumour incidence in wild-type and p53-deficient mice by the classical multistage single-path model.

### Multistage multipath model

Recently, several authors have considered an extension of the multistage model that allows for the development of any type of tumour by alternative multistage pathways involving different sets of genes (Tan, 1991; Sherman and Portier, 1994). This concept is

**Table 3** Predicted tumour development by the p53-mediated pathway in wild-type and p53-deficient mice on a 5-stage model for p53-mediated tumorigenesis with 20% of all tumours in wild-type mice developing by the p53-mediated route and 80% by p53-independent pathways

| Genotype | Five-stage model (Mutation rate = $7.5 \times 10^{-5} \pm 1.5 \times 10^{-5}$) p53-mediated tumours per mouse | |
|---|---|---|
| | 16 weeks | 80 weeks |
| p53+/+ | 0 | 0.038 |
| p53+/– | 0.006 | 0.83 |
| p53–/– | 0.863 | 193 |

© Cancer Research Campaign 1998

**Figure 5** General structure of the multigate model with a two-stage mutational rate-modifier pathway and with three gate-pass events required from stem cell to malignancy

**Table 4** Predicted tumour development in wild-type and p53-deficient mice on a three-gate model with two-stage modifier (p53 inactivation) pathway. On this model tumours may arise by the same gateway with or without p53 inactivation; the proportion having inactivated p53 is shown in the second column

| Genotype | Three-gate model with two-stage modifier ($\lambda = 10^{-4} \pm 2 \times 10^{-5}$, $\mu = 10^{-5} \pm 2.5 \times 10^{-6}$, $k = 10$) Tumours/mouse | | Proportion of all tumours developing by 80 weeks with inactivated p53 |
|---|---|---|---|
| | 16 weeks | 80 weeks | |
| p53+/+ | 0.0004 | 0.10 | 0.055 |
| p53+/– | 0.0015 | 0.91 | 0.92 |
| p53–/– | 0.56 | 375 | 1.00 |

illustrated in Figure 3. In the present context, this means that a proportion of tumours in wild-type mice develop by pathways that are independent of p53 status and would have the same probability of occurrence in p53-deficient mice. To investigate this, we have rerun the multistage single path model on the assumption that only a fraction, *f*, of the tumours developing in wild-type mice have arisen by the p53-mediated pathway. It is then only the p53-mediated tumours whose frequency is increased in the p53-deficient genotypes. We have run three-, four- and five-stage models for a range of *f*-values and observed similarity with the experimental data only for the five-stage model with $f = 0.2$ (i.e. 20% of all tumours in wild-type mice developing by the p53-mediated pathway). For $f > 0.2$, an excess of tumours are predicted for p53–/– mice and for $f < 0.2$ a deficit of tumours in the p53+/– genotype (Figure 4). The data for p53-mediated tumours for $f = 0.2$ are shown in Table 3.

The detailed age dependence of tumour development cannot be computed on this model as the process of tumour development is only defined mechanistically for the p53-mediated pathway – the non-p53-mediated path has unspecified structure and parameters. For this reason, Table 3 shows only the tumours developing by the p53-mediated route; in the p53+/– and p53–/– (but not wild-type) genotypes these will be the majority of tumours. Table 3 shows that more reasonable tumour numbers may be calculated for each of the genotypes, considering that the experimental latency time for p53+/– mice is about 71 weeks and for p53–/– mice is about 16 weeks. This means that the multistage multipath model does seem capable of being reconciled with most of the data. The need to postulate alternative, as yet undefined pathways, is an unsatisfactory feature of the model, as is the inability to compute age-dependence curves but does not mean this process could not occur in biological reality. In the sections that follow, we will consider a different model that incorporates recent thinking about the role of p53 and that appears to accommodate the data in a more natural way.

### A multigate model with two-stage modification of mutation rate

In each of the preceding models, mutations occurred independently and the rate of mutation at any stage was unaffected by mutations that had already occurred at other stages. However, it is a current hypothesis, termed by Lane (1992) the 'guardian of the genome' concept, that p53 inactivation results in generally increased mutation rates, i.e. wild-type p53 acts to confer genetic

© Cancer Research Campaign 1998 

**Figure 6** Predicted pattern of time of tumour development for mice of three genotypes for the multigate model with two modifier stages (mutation rate = $10^{-4}$ per cell division) and three gate-pass events (mutation rate = $10^{-5}$ per cell division) and a mutation rate modifying factor of 10. The vertical broken lines in the diagrams show median latency of experimental tumours [averaged data from Donehower et al (1995); Table 1]

**Figure 7** Predicted proportion of p53 inactivation-associated tumours appearing as a function of mouse age in the p53 wild-type and heterozygous genotypes. ●, p53 (+ –) mice; ▼, p53 (+/+) mice stability. Similarly, on this model, p53 inactivation could lead to increased survival of cellular mutants that would otherwise have died (e.g. as a result of p53-dependent apoptosis). We now wish to consider how this idea may be incorporated in stochastic modelling and how well the model accommodates the data. To do this, it may be useful to distinguish between mutations that are directly or indirectly tumorigenic.

Directly tumorigenic mutations, whether these are oncogene activations or inactivations of tumour-suppressor genes, are 'enabling' or obligatory events that must accumulate to a minimum number, or possibly to one of several alternative configurations, for malignant transformation of the affected cell. We may consider that tumorigenesis requires a number of regulatory 'gates' to be passed and that a tumorigenic mutation of a direct type alters a gateway gene and corresponds to a gate-pass event. The gate-pass events are the stages of the multistage model. However, indirect mutations are not enabling events in themselves but modifiers of the tumorigenic mutation rate. This leads to a multigate model of tumorigenesis with mutation rates under the control of rate-modifier genes. Mutated rate-modifier genes lead to altered mutation rates in gateway genes. A similar concept has been proposed by Loeb (1991), who has argued for the existence of a 'mutator phenotype' and by Sherman and Portier (1994), who have termed such a process a 'multihit' model to distinguish it from multistage. Very recently, Kinzler and Vogelstein (1997) have proposed a distinction between 'gatekeeper' and 'caretaker' genes whose inactivation contributes to tumorigenesis, either directly or indirectly. However, the properties of such models have not yet been explored and it has not previously been applied to p53-mediated tumorigenesis. The structure of a three-gate model, associated with a two-stage modifier or 'caretaker' (p53-mediated) pathway is depicted in Figure 5.

A feature of the model is that mutation of the gateway genes alone (without modifier gene mutations) may lead to malignant transformation, whereas modifier mutations cannot achieve transformation without gate-pass events. Therefore, only a proportion of tumours developing by this gateway will be associated with modifier mutations. This proportion will depend on the numbers of genes involved in the gateway and in the modifier pathway, and on rates of mutation of modifier genes and of gateway genes, when modifier mutations have or have not occurred. Generally, the proportion of modifier-associated mutations will be low unless there are more gates than modifier stages or unless the modifier genes are themselves more prone to mutation than the gateway genes. The new model is more complex than the classical multistage model; an investigation of its properties will be reported in detail elsewhere. Here, we wish to establish whether this type of model can account for tumour incidence data in p53-deficient mice.

To apply these concepts to tumorigenesis in p53-deficient mice, we propose that p53 is a rate-modifying (caretaker) gene whose inactivation requires two stages in wild-type mice and one-stage inactivation in p53+/– mice; of course the gene is already inactivated in p53–/– mice. In each genotype, the same number of gateway gene mutations is required. Gateway genes have a baseline mutation rate $\mu$ when p53 function is maintained, which is increased to mutation rate $k\mu$ when p53 function is lost. The mutation rate for two-stage loss of p53 function is assigned the independent value $\lambda$. Computer simulations have been carried out for a range of values of the number of gates in the model, for a range of values of the mutation rates of the modifier genes and (independently) the mutation rate of the gate-pass genes, and a range of values of the modifying factor (the scaling factor for gate-pass mutation rate). We have observed that better agreement with the data is found when the modifier gene mutation rate is higher than that of the gate-pass genes (implying that p53 is itself relatively genetically unstable). The modifying factor then has a value around ten. Table 4 and Figure 6 show predicted data for three mouse genotypes for this case. In support of this hypothesis, some evidence suggests that after the loss of the first p53 allele, loss of the second allele occurs more easily (Harvey et al, 1993). Further experiments will be needed to confirm this possibility.

It can be seen that realistic predictions of tumour numbers in the three genotypes can now be achieved. Notice that the mean tumour number/mouse is now slightly less than 1 for p53+/– and p53–/– mice by 80 and 16 weeks respectively, although large tumour numbers would still be predicted for p53–/– mice by 80 weeks; however, no such mice will survive to this time. The model parameters are not uniquely defined by the available data and other combinations of gate number and mutation and modifying factors may be possible. We will present a full description of the mathematical properties of the multigate model in a forthcoming publication.

The model also provides predictions of the proportions of tumours that occur in each genotype in association with p53 inactivation; these are shown for tumours accumulated by 80 weeks in the last column of Table 4. We have also computed the proportion

 © Cancer Research Campaign 1998

of p53 inactivation-associated tumours appearing as a function of mouse age in the wild type and p53+/– genotypes and have observed that this proportion shows a tendency to increase with age (Figure 7), implying that p53 inactivated tumours will be relatively over-represented among late-occurring tumours. Comprehensive experimental data on this have not yet been reported for p53-deficient mice. When this data is available, it will provide a more stringent requirement that should enable a test of the model and allow the parameters to be specified more precisely.

### DISCUSSION AND CONCLUSIONS

The analysis has demonstrated a fundamental problem in the application of the classic multistage model to spontaneous tumorigenesis in p53-deficient mice. On a multistage model, with a single pathway of tumorigenesis, the reduction in stage number by one, resulting from germ line inheritance of one of the tumorigenic mutations, without change of mutation rate, results in a marked increase in predicted tumour frequency. Transgenic mice have provided a unique opportunity to test the prediction that the inheritance of two tumorigenic mutations (inactivated p53 alleles), corresponding to a reduction in stage number by two, would produce an astronomical number of tumours per mouse. Our analysis shows that this prediction applies for up to five stages being required for tumorigenesis in wild-type mice.

This difficulty has been recognized previously. In 1990, Vogelstein commenting on tumour incidence in human Li–Fraumeni patients posed the question 'Why don't these patients develop more tumours?' and commented 'Given the diverse tumours occurring in Li–Fraumeni patients it would seem that many human cell types are susceptible to the effect of inherited p53 mutations; yet the median age of tumour development is over 30 years and the median number of lifetime tumours is less than two' (Vogelstein, 1990). Vogelstein's paradox also occurs for the double-defect p53–/– mice, which still show no more than a few tumours per mouse, although thousands would be predicted. This analysis has identified two categories of explanation for this paradox. The first of these, the multistage multipath model invokes a p53-independent pathway that exists in parallel with a p53-mediated route of tumorigenesis. Only the latter route is enhanced in p53-deficient genotypes. On this type of model, the mutation rates are independently fixed and these inherent rates are not changed by p53 inactivation. We have found that a p53-mediated five-stage pathway that provides 20% of the tumours in wild-type mice is consistent with the data.

The second mechanism, which we have called the multigate model, postulates a single pathway (or gateway) with several gate-pass events (obligatory mutations) occurring at a rate that depends on p53 status. We have not yet fully explored this category but have observed that the data can be accommodated by a three-gate model in which the gate-pass mutation rate is amplified by a factor of about 10 when both p53 alleles are inactivated. It is also possible to envisage combined models (multigate multipath) but the currently available data do not require a combined model to be invoked.

It should again be noted that the present model does not distinguish a higher mutation rate in itself from higher probability of survival of mutants. It would therefore be consistent with a role for p53 in DNA damage-mediated apoptosis provided the apoptosis rate remained low (see below).

The multigate model differs from the multistage/multipath model in postulating that p53 inactivation has a rate-modifying role in a tumorigenesis pathway that can nevertheless proceed independently of p53. Suppose, for example, that inactivation of both Rb alleles are two enabling (gate-pass) genetic events in a particular tumour type. Then we expect that some tumours will be Rb doubly mutant (with intact p53) others will be Rb doubly mutant with inactivated p53. However, no tumours will be found to have suffered only p53 inactivation. The inactivation of p53 would therefore appear to be 'optional' in this mechanism of tumorigenesis. The multistage multipath model requires that if p53 is implicated in a tumorigenic pathway then any alternative pathway not involving p53 will instead have to involve some other genetic events (e.g. some genetic event additional to, or as an alternative to, the Rb inactivation considered in the example), i.e. p53 inactivation fulfils a role that is not 'optional' and would have to be replaced. It is also a feature of the multigate model that p53 inactivation should precede at least one of the gate-pass events, whereas on the multistage model p53 inactivation could just as easily come last.

Of course, the main difference between the models is that the multigate model is essentially a genetic instability model and requires that p53 inactivation be a destabilizing event. Experimental evidence on this is not wholly consistent at present, with some workers reporting a significant increase in the mutation rate at a particular locus (Havre et al, 1995; Xia et al, 1995) and no difference being reported at other loci (Sands et al, 1995). In addition it is possible that the tumorigenic events that are enhanced by p53 inactivation correspond to chromosome abnormalities (Bouffler et al, 1995) or gene amplification (Livingstone et al, 1992; Yin et al, 1992) or other heritable events rather than traditional point mutations; also, that the influence of p53 inactivation is confined to a restricted set of genes rather than being across the genome. It is also possible that the main impact of p53 inactivation will be in relation to the processing of DNA damage and that its role will be seen more clearly when mice of differing genotypes are subjected to graded doses of DNA-damaging agents.

These analyses have focused on the stage number and mutation rates as major determinants of the tumorigenesis process. The models considered have not assumed any proliferative advantage of intermediate (premalignant) cells. However, it is possible that intermediate cell proliferation could play a significant role in tumorigenesis. Moolgavkar and Luebeck (1992) have proposed a model for the role of the APC gene in colon cancer that entails APC-mediated control of intermediate cell proliferation. A similar role for p53 could be considered. In addition, Bodmer and Thomlinson (1996), in a recent model, have emphasized the p53-mediated control of apoptosis (preferential elimination of mutant cells) rather than mutation rate itself. This is approximately equivalent to the multigate model assumption of modification of mutation rate if the apoptosis rate is ordinarily low (reduced apoptosis in p53-deficient cells allowing the improved survival of mutants) but a high rate of apoptosis in wild type cells would lead indirectly to a proliferative advantage of p53 null cells (by imposing a higher loss rate on p53+/+ and p53+/– cells). These variations have not yet been fully explored in our analysis. Nevertheless, it seems clear that the single-pathway multistage model for p53-mediated tumorigenesis will have great difficulty in accommodating the experimental data, and that alternatives to this model must be sought.

We will extend the analysis to radiation-induced tumorigenesis in p53-deficient mice, for which some experimental data have already been reported (Kemp et al, 1994). We will also extend the model to deal with the development of tumours of several different types (e.g. lymphomas, sarcomas) that may have different mutation

© Cancer Research Campaign 1998 

rates or stage (gate) number or differing stem cell kinetics. We expect that stochastic modelling of tumorigenesis in p53-deficient mice will contribute to an understanding of tumorigenesis in human Li–Fraumeni patients as well as to the role of p53 in human cancer more generally. The approach taken here should also prove useful for analysis of tumorigenesis in other transgenic mouse models especially in which, as in the MSH2-deficient (mismatch repair deficient) transgenic model (Reitmar et al, 1995), the genetic defect would be expected to act as a modifier of putative gate-pass events (i.e. probable inactivation of a caretaker gene). More generally, the analysis may help in the distinction between gatekeeper and caretaker genes in many tumorigenic pathways.

### REFERENCES

Bodmer WF and Thomlinson I (1996) Population genetics of tumours. *CIBA Foundation Symposia* **197**: 181–189

Bouffler SD, Kemp CJ, Balmain A and Cox R (1995) Spontaneous and ionizing radiation-induced chromosomal-abnormalities in p53-deficient mice. *Cancer Res* **55**: 3883–3889

Donehower LA, Harvey M, Slagle BL, McArthur MJ, Montgomery CA, Butel JS and Bradley A (1992) Mice deficient for p53 are developmentally normal but susceptible to spontaneous tumours. *Nature* **356**: 215–221

Donehower LA, Harvey M, Vogel H, McArthur MJ, Montgomery CA, Park SH, Thompson T, Ford RJ and Bradley A (1995) Effects of genetic background on tumorigenesis in p53-deficient mice. *Mol Carcinogen* **14**: 16–22

Fowlis DJ and Balmain A (1993) Oncogenes and tumour suppressor genes in transgenic mouse models of neoplasia. *Eur J Cancer* **29A**: 638–645

Havre PA, Yuan JL, Hedrick L, Cho KR and Glazer PM (1995) p53 inactivation by HPV16 E6 results in increased mutagenesis in human cells. *Cancer Res* **55**: 4420–4424

Harvey M, McArthur MJ, Montgomery CA, Butel JS, Bradley A and Donehower LA (1993) Spontaneous and carcinogen-induced tumorigenesis in p53-deficient mice. *Nature Genet* **5**: 225–229

Harvey M, Sands AT, Weiss RS, Hegi ME, Wiseman RW, Pantazis P, Giovanella BC, Tainsky MA, Bradley A and Donehower LA (1993) In vitro growth characteristics of embryo fibroblasts isolated from p53-deficient mice. *Oncogene* **8**: 2457–2467

Hollstein M, Sidransky D, Vogelstein B and Harris CC (1991) p53 mutations in human cancers. *Science* **253**: 49–53

Hursting SD, Perkins SN and Phang JM (1994) Calorie restriction delays spontaneous tumorigenesis in p53-knockout transgenic mice. *Proc Natl Acad Sci USA* **91**: 7036–7040

Jacks T, Remington L, Williams BO, Schmitt EM, Halachmi S, Bronson RT and Weinberg RA (1994) Tumour spectrum analysis in p53-mutant mice. *Current Biol* **4**: 1–7

Kemp CJ, Wheldon T and Balmain A (1994) p53-deficient mice are extremely susceptible to radiation-induced tumorigenesis. *Nature Genet* **8**: 66–69

Kinzler KW and Vogelstein B (1997) Gatekeepers and caretakers. *Nature* **386**: 761–763

Knudson AG (1971) Mutation and cancer: Statistical study of retinoblastoma. *Proc Natl Acad Sci USA* **68**: 820–823

Knudson AG (1996) Hereditary cancer – 2 Hits revisited. *J Cancer Res Clin Oncol* **122**: 135–140

Lane DP (1992) p53, guardian of the genome. *Nature* **358**: 15–16

Lane DP (1994) The regulation of p53 function – Steiner-award lecture. *Int J Cancer* **57**: 623–627

Livingstone LR, White A, Sprouse J, Livanos E, Jacks T and Tlsty T (1992) Altered cell cycle arrest and gene amplification potential accompany loss of wild-type p53. *Cell* **70**: 923–935

Loeb LA (1991) Mutator phenotype may be required for multistage carcinogenesis. *Cancer Res* **51**: 3075–3079

Mao JH and Wheldon TE (1995) A stochastic model for multistage tumorigenesis in developing and adult mice. *Math Biosci* **129**: 95–110

Moolgavkar SH and Knudson AG (1981) Mutation and cancer: A model for human carcinogenesis. *J Natl Cancer Inst* **66**: 1037–1052

Moolgavkar SH and Luebeck EG (1992) Multistage carcinogenesis: Population-based model for colon cancer. *J Natl Cancer Inst* **84**: 610–618

Moolgavkar SH and Venzon D (1979) Two-event models for carcinogenesis: Incidence curves for childhood and adult tumours. *Math Biosci* **47**: 55–77

Purdie CA, Harrison DJ, Peter A, Dobbie L, White S, Howie SEM, Salter DM, Bird CC, Wyllie AH, Hooper ML and Clarke AR (1994) Tumour incidence, spectrum and ploidy in mice with a large deletion in the p53 gene. *Oncogene* **9**: 603–609

Reitmar AH, Schmits R, Ewel A, Bapat B, Redston M, Mitri M, Waterhouse P, Mittrucker HW, Wakeham A, Liu B, Thomason A, Griesser H, Gallinger S, Ballhausen WG, Fishel R and Mak TW (1995) MSH2 mice are viable and susceptible to lymphoid tumours. *Nature Genet* **11**: 64–70

Renan MJ (1993) How many mutations are required for tumorigenesis? Implications from human cancer data. *Mol Carcinogen* **7**: 139–146

Sands AT, Suraokar MB, Sanchez A, Marth JE, Donehower LA and Bradley A (1995) p53 deficiency does not affect the accumulation of point mutations in a transgene target. *Proc Natl Acad Sci USA* **92**: 8517–8521

Sherman CD and Portier CJ (1994) The multipath/multistage model of carcinogenesis. *Inform Biom Epidemiol Med Biol* **25**: 250–254

Tan WY (1991) *Stochastic Models of Carcinogenesis.* Marcel Dekker: New York

Vogelstein B (1990) A deadly inheritance. *Nature* **348**: 681–682

Vogelstein B and Kinzler KW (1993) The multistep nature of cancer. *Trend Genet* **9**: 138–141

Xia F, Wang X, Wang YH, Tsang NM, Yandell DW, Kelsey KT and Liber HL (1995) Altered p53 status correlates with differences in sensitivity to radiation-induced mutation and apoptosis in two closely related human lymphoblast lines. *Cancer Res* **55**: 12–15

Yin Y, Tainsky MA, Bischoff FZ, Strong LC and Hall GM (1992) Wild-type p53 restores cell cycle control and inhibits gene amplification in cells with mutant p53 alleles. *Cell* **70**: 937–948

### APPENDIX: MATHEMATICAL DEVELOPMENT OF TUMORIGENESIS MODELS

### Model 1: multistage single path model

This model (Figure 1) is a *k*-stage model of tumorigenesis with cell proliferation and cell loss in all stages. Let $X_0(t)$, $X_1(t)$, ..., $X_{k-1}(t)$ and $X_k(t)$ represent the number of stem cells, one-stage mutants, ..., *k*–1-stage mutants and malignant cells generated from stem cells by time *t* respectively. At time $t = 0$, $X_0(0) = 1$, $X_j(t) = 0$, ($j = 1, 2, ..., k$), and $T(0) = 0$. In a small time interval $[t, t + \Delta t]$, a *j*-stage mutant may:

(a) divide into two *j*-stage mutants at rate $b_j(t)\Delta t$+o($\Delta t$);
(b) die (or differentiate) at rate $d_j(t)\Delta t$+o($\Delta t$);
(c) divide into one *j*-stage mutant and one $j + 1$-stage mutant at rate $\mu_j\Delta t$+o($\Delta t$). $j = 0, 1, ..., k-1$ where a 0-stage mutant is a stem cell and a *k*-stage mutant is a tumour cell, or
(d) stay unchanged.

All cells go through the above processes independently of other cells. For the application to tumorigenesis in both wild-type and p53-deficient mice, the single pathway is deemed to require inactivation of both p53 alleles as obligatory stages.

### Model 2: multistage multipath model

Figure 3 displays a two-pathway multistage model of tumorigenesis. There are two possible routes for a normal cell to be transformed into a malignant cell: (a) a p53-mediated route and (b) a p53-independent route. A tumour develops independently by any of the two pathways. The essential features of each pathway are as described in model 1. The total rate of tumorigenesis is the sum of the tumorigenesis rates occurring by each of the two pathways.

### Model 3: multigate model with multistage modification of mutation rate

In this model, p53 inactivation at both alleles acts as a modifier of the rate of mutation of the tumorigenic states (gates). Figure 5 shows a three-gate/two-stage model as an example. Let $X_{01}(t)$,

© Cancer Research Campaign 1998

**Figure 8** Predicted number of normal stem cells ($X_0$), 1-stage mutants ($X_1$) and 2-stage mutants ($X_2$) by the three-stage model shown (**A**) over a 0–30 day time scale, (**B**) over 0–600 day time scale (mouse lifespan). The family of curves in each case correspond to computer simulation for individual mice

$X_{02}(t)$, $X_{03}(t)$, $X_{11}(t)$, $X_{12}(t)$, $X_{13}(t)$, $X_{21}(t)$, $X_{22}(t)$, $X_{23}(t)$ and $Y(t)$ represent the numbers of stem cells with p53+/+ (S/p53+/+), stem cells with p53+/– (S/p53+/–), stem cells with p53–/– (S/p53–/–), gate-1 mutants with p53+/+ (G1/p53+/+), gate-1 mutants with p53+/– (G1/p53+/–), gate-1 mutants with p53–/– (G1/p53–/–), gate-2 mutants with p53+/+ (G2/p53+/+), gate-2 mutants with p53+/–, (G2/p53+/–), gate-2 mutants with p53–/– (G2/p53–/–), and malignant cells generated from stem cells by time $t$ respectively. At time $t = 0$, $X_{01}(t)$, = 1, $X_{02}(t) = 0$, $X_{03}(t) = 0$, $X_{11}(t) = 0$, $X_{12}(t) = 0$, $X_{13}(t) = 0$, $X_{21}(t) = 0$, $X_{22}(t) = 0$, $X_{23}(t) = 0$ and $Y(t) = 0$. These assumptions allow a table of transition probabilities to be constructed (not shown) that defines the mathematical structure of the model.

### Computer simulation

The stochastic simulation process used can be described as the time-slice approach, in which the tumorigenic process is viewed as

© Cancer Research Campaign 1998

changing in all of its aspects over time. Its status is updated, in units of one cell cycle time, until a prescribed amount of time has elapsed. In our studies, we suppose the cell cycle time is not influenced by the mutational events.

For normal stem cells and mutants, the distribution of the number of cells was computed without following the fate of every cell: homogeneous populations of normal stem cells or mutants were treated as groups. For such groups only the number of cells were updated, using a multinominal random number generator, but using Poisson and normal random number generators to provide approximation when the number of cells is very large. Further details are given by Mao and Wheldon (1995). Malignant cells that have arisen from normal stem cells are followed individually. Some samples are shown in Figure 8. This shows the occurrence of fluctuations in the growth pattern at small cell number, due to the stochastic nature of both the growth and mutation process. These fluctuations are smoothed out as the cell number becomes larger.

The method for estimating the parameters in the models, which involves matching the tumour-free survival distribution of the experimental data with simulated data by a Monte Carlo method, is as follows:

Partition the time interval $[0, t]$ by $I_j = [t_{j-1}\ t_j), j = 1, \ldots, m-1$, and $I_m = [t_{m-1}, t_m]$, in which $t_j = j^*\ \Delta t$ and $m^*\ \Delta t = t$ with $t = t_m$ (in our studies, $\Delta t = 7$ days, $m = 86$). Let $N_j$ and $n_j$ be the number of mice that acquired tumour during $I_j$ for the simulated and experimental data respectively. The parameters are estimated by minimizing the chi-square statistic

$$\chi^2 = \Sigma^m_{j=1} \frac{(N_j - n_j)^2}{N_j + n_j}$$

In model 1, the values of the growth and death rate parameters were prescribed by

$$b_j(t) = \begin{cases} 0.564 & t \leq 13 \text{ days,} \\ 0.425e^{-0.231(t-13)} + 0.139 & t \geq 13 \text{ days,} \end{cases}$$

$$d_j(t) = 0.139\ (j = 1, 2, \ldots, k-1),$$

and were chosen to provide reasonable growth kinetics for mouse development from conception onwards. The same values were used for models 2 and 3. The mutation rate was estimated to match the tumorigenesis rate in wild-type mice.

Besides the mutation rate, in model 2, the fraction f of the tumours developing by p53-mediated route in wild mice, and in model 3, the modifier factor $k$, was estimated.

© Cancer Research Campaign 1998